# Autologous Genetically Enriched Leucoconcentrate in the Preventive and Acute Phases of Stroke Treatment in a Mini-Pig Model

**DOI:** 10.3390/pharmaceutics14102209

**Published:** 2022-10-17

**Authors:** Zufar Safiullov, Andrei Izmailov, Mikhail Sokolov, Vage Markosyan, Grayr Kundakchan, Ravil Garifulin, Maksim Shmarov, Boris Naroditsky, Denis Logunov, Rustem Islamov

**Affiliations:** 1 The Department of Histology, Cytology and Embryology, Kazan State Medical University, 420012 Kazan, Russia; 2 The National Research Center for Epidemiology and Microbiology Named after Honorary Academician N.F. Gamaleya of the Ministry of Health of the Russian Federation, 123098 Moscow, Russia

**Keywords:** autologous genetically enriched leucoconcentrate, chimeric adenoviral vector, vascular endothelial growth factor, glial-cell-line-derived neurotrophic factor, neural cell adhesion molecule, personalized cell-mediated gene therapy, ischaemic stroke, mini-pig

## Abstract

The natural limitations of regeneration in the CNS are major problems for the treatment of neurological disorders, including ischaemic brain strokes. Among the approaches being actively developed to inhibit post-ischaemic negative consequences is the delivery of therapeutic genes encoding neuroprotective molecules to the brain. Unfortunately, there are currently no proven and available medicines that contain recombinant human genes for the treatment of ischaemic cerebral stroke. Of particular interest is the development of treatments for patients at risk of ischaemic stroke. In the present study, we propose a proof of concept for the use of an autologous, genetically enriched leucoconcentrate temporally secreting recombinant vascular endothelial growth factor (VEGF), glial-cell-line-derived neurotrophic factor (GDNF) and the neural cell adhesion molecule (NCAM) for the treatment of stroke. In a mini-pig ischaemic stroke model, genetically enriched leucoconcentrate was infused 4 h after surgery (gene therapy in acute phase) or 2 days before stroke modelling (preventive gene therapy). On day 21, after the stroke modelling, the post-ischaemic brain recovery was examined by morphologic and immunofluorescence analysis. The benefits of treating a stroke with genetically enriched leucoconcentrate both for preventive purposes and in the acute phase were confirmed by an improved performance in behavioural tests, higher preservation of brain tissue and positive post-ischaemic brain remodelling in the peri-infarct area. These results suggest that the employment of autologous leucocytes enabling the temporary production of the recombinant therapeutic molecules to correct the pathological process in the CNS may be one of the breakthrough approaches in gene therapy.

## 1. Introduction

Strokes are one of the leading causes of death worldwide, and the lack of effective therapies for acute cerebrovascular injuries is one of the most pressing medical problems [1]. To date, there are no therapeutic protocols that can be used to reduce the effects of a post-ischaemic brain injury and stimulate revascularisation and neuro-regeneration after stroke in practical medicine. The difficulties in treating stroke are due to drastic necrosis during the first few minutes in the ischaemic core, followed by apoptosis in the surrounding penumbra for a 3–6 h period (therapeutic window) and degeneration in the ischemic area for several weeks [2]. The current urgent treatment aims to remove the clot and to restore the cerebral blood flow. However, for the preservation of the brain tissue, the stroke pathophysiology suggests that the therapeutic impacts not only on ischaemia but also on excitotoxicity be addressed, along with apoptosis and neuroinflammation to prevent brain cell death [3]. To date, cell [4,5] and gene [6,7] therapies are proposed as promising therapeutic strategies for stroke treatment.

It is well known that, during neuro-ontogenesis, the survivability and migration of neural progenitor cells, directed axon growth and synaptogenesis are realised through neurotrophic and growth factors produced in certain neurons or vascular and glial cells. Thus, the general principles of neuro-ontogenesis may also apply to neuro-regeneration. Moreover, the redeployment of the embryonic gene regulatory network during regeneration [8] also indicates that one could use the genes involved in neurogenesis to stimulate neuro-regeneration in adults with CNS disorders. The delivery of the recombinant cDNA encoding neurotrophic factors, such as nerve growth factor (NGF), vascular endothelial growth factor (VEGF), brain derived growth factor (BDNF), glial-cell-line-derived neurotrophic factor (GDNF), basic fibroblast growth factor (BFGF) and ciliary neurotrophic factor (CNTF), demonstrated positive therapeutic effects on the ischaemic brain [7,9].

Recently, to examine stroke gene therapy in the acute phase [10] and preventive [11] treatment in a rat model, we employed human umbilical cord blood mononuclear cells (UCB-MC) for the tandem delivery of three therapeutic genes encoding VEGF, GDNF, and the neural cell adhesion molecule (NCAM). We showed the positive effects of the gene-modified UCB-MC on the negative consequences of the ischaemia-induced brain injury. However, in practical medicine, the use of the UCB-MC is restricted not only due to ethics and possible immunogenic problems, but also because it is seriously limited by the small amounts of UCB-MC that can be acquired from one donor and by the deficiency of these types of cells in general. In view of this, we developed a new personalised precision cell-mediated (ex vivo) gene therapy approach based on the intravenous administration of a patient’s leucoconcentrate, simultaneously transduced with three chimeric adenoviral vectors (Ad5/35F) carrying cDNA encoding VEGF, GDNF, and NCAM in equal ratios for the treatment of spinal cord injuries in a mini-pig model [12,13].

Many of the studies testing the new approaches to stroke treatment have been performed on rodent models, which have limited translational potential. The pathophysiology and pathomorphology of stroke in rodents with lissencephalic brains are not fully comparable with brain injuries in human stroke [14]. Therefore, preclinical stroke studies need to be performed on large mammals that have neuroanatomical, physiological and biochemical characteristics that are close to those of a human. Pigs not only have a gyrencephalic brain and white matter volume similar to that of humans [14], but they are also genetically closer to humans than mice [15], which suggests the practicality of using a porcine model of ischaemic stroke for the discovery of new approaches to stroke treatment. Thus, swine stroke models are considered to have translational advantages compared to rodents, dogs, and non-human primates.

**Figure 1:**
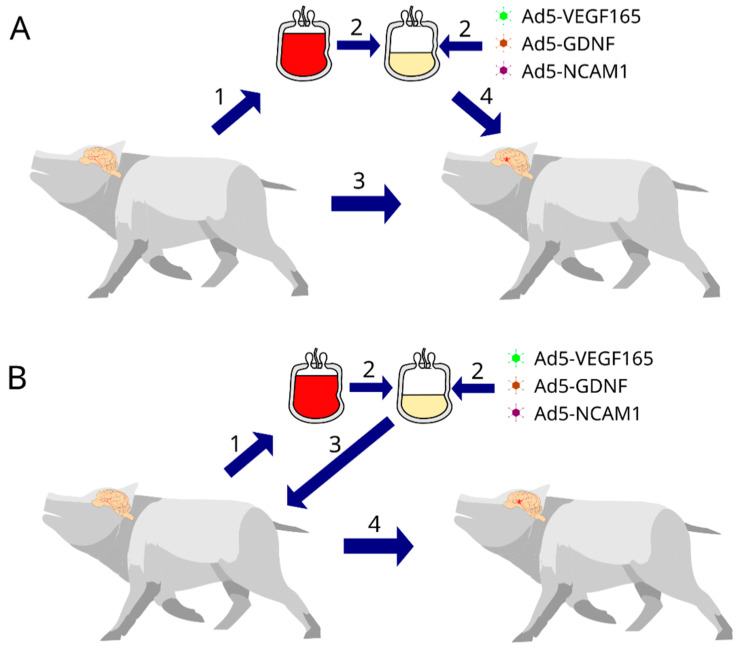
Study design. (**A**) cell-mediated gene therapy in acute phase of stroke: (1) collection of 50 mL of venous blood 14 h before stroke modelling; (2) preparation of the genetically enriched leucoconcentrate; (3) stroke modelling; (4) intravenous infusion of the autologous genetically enriched leucoconcentrate 4 h after surgery. (**B**) preventive cell-mediated gene therapy for stroke: (1) collection of 50 mL of venous blood; (2) preparation of the genetically enriched leucoconcentrate; (3) intravenous infusion of the autologous genetically enriched leucoconcentrate; (4) stroke modelling 2 days after the genetically enriched leucoconcentrate infusion.

In the present mini-pig study, we developed a protocol for simulating moderate ischemic cerebral stroke and evaluated the efficacy of the autologous, genetically enriched leucoconcentrate (GEL) producing recombinant VEGF, GDNF, and NCAM in the acute phase (intravenous infusion 4 h after stroke modelling) and preventive phase (intravenous infusion of GEL 2 days before stroke modelling) of stroke gene therapy (Figure 1).

## 2. Material and Methods

### 2.1. Animals

Miniature Vietnamese pot-bellied 8-month-old female pigs (25–30 kg; *n* = 16) were obtained from the Kazan State Academy of Veterinary Medicine by N.E. Bauman (Kazan, Russia). Throughout the investigation, the mini-pigs were kept with one per housing area under controlled temperatures (24–25 °C), air conditioning, a 12 h light/dark cycle, and properly organised feeding. All experimental procedures were approved by the Kazan State Medical University Animal Care and Use Committee (approval No. 5, dated 26 May 2020).

### 2.2. Autologous Genetically Enriched Leucoconcentrate

Genetically enriched leucoconcentrate (GEL) was prepared according to our original protocol, as described previously [13]. With the mini-pigs under anaesthesia following an intramuscular injection of tiletamine/zolazepam (Zoletil 100, Virbac Laboratoires, Carros, France; 10 mg/kg) and xylazine (Xyla, Interchemie werken ‘De Adelaar’ B.V., Castenray, the Netherlands; 40 mg/kg), 50 mL of peripheral blood was collected from *V. subclavia*. The blood was collected in a plastic blood bag of 250 mL with anticoagulant CPDA-1 (Green Cross, Yongin-si, Korea) (the volume of preservative in each bag was 35 mL). The leucoconcentrate was prepared in the blood bag according to our original protocol, which sequentially includes the sedimentation of erythrocytes with 6% hydroxyethyl starch, followed by centrifugation at 34× *g* for 10 min at 10 °C (DP-2065 R PLUS, Centrifugal Presvac RV; Presvac, Buenos Aires, Argentina) and washing with saline. The supernatant was discarded from the bag, and the remaining solution containing white blood cells (WBC) in the bag was considered to be the leucoconcentrate.

Chimeric (Ad5/35F) viral vectors carrying cDNA encoding VEGF, GDNF, NCAM and reporter green fluorescent protein (GFP) were developed based on the human adenovirus serotype 5 with a fibre derived from adenovirus serotype 35 (Ad5/35) [13]. The transduction of the obtained leucoconcentrate was conducted using a blood container with an MOI (multiplicity of infection) equal to 10 based on the WBC count and the titres of the adenoviral vectors. An equal ratio of each chimeric adenoviral vector, Ad5/35-VEGF165 (2.0 × 10^9^ PFU/mL), Ad5/35-GDNF (7.0 × 10^10^ PFU/mL), and Ad5/35-NCAM1 (5.0 × 10^10^ PFU/mL), was injected into the blood bag, and the leucoconcentrate was transduced for 12 h under constant rocking on a shaker (ELMI) at room temperature (21–22 °C). After that, 200 mL of sterile saline was added to the blood bag, and it centrifuged at 1000 rpm at 10 °C for 10 min. The supernatant (saline containing cell fragments and free viral particles) was extracted from the container, so that approximately 30 mL of solution remained in the container, including the cell suspension, a leucoconcentrate enriched with genetic material (GEL-VGN). The reporter GEL-GFP was prepared using Ad5/35-GFP (2.5 × 10^9^ PFU/mL). The efficacy of the therapeutic (VEGF165, GDNF, NCAM1) and reporter (GFP) transgenes’ expression was confirmed as described previously [13]. In the present study, the GFP expression in the WBCs in the mini-pig brain was confirmed using fluorescent microscopy 7 days after the intravenous auto-infusion of GML-GFP into the mini-pigs with stroke.

### 2.3. Ischaemic Stroke Modelling

In clinical practice, middle cerebral artery (MCA) occlusion is the primary cause of cerebral damage in humans. Therefore, the MCA is the main target of in vivo stroke models [16]. The peculiarities of the cerebral vascular anatomy of pigs, such as the presence of two to four MCAs on each side of the brain and the involvement of the anterior and posterior cerebral arteries in the circulation in the circle of Willis, present certain difficulties in reaching a reproducible ischemic cerebral infarction [14]. In the study, we employed the protocol which includes the sequential occlusion of the right common carotid artery and the left MCA. The animals were intramuscularly anaesthetised with 3 mg/kg Zoletil^®^100 (Virbac Sante Animale, Carros, France) and subsequently connected to an inhalation anaesthesia apparatus (Minor Vet Optima, Zoomed, Moscow, Russia) through which 2.0–2.5% isoflurane (Laboratorios Karizoo, S.A., Barcelona, Spain) mixed with oxygen was administered. All surgical manipulations were performed in accordance with the aseptic and antiseptic rules. In the first step, the right common carotid artery was ligated to reduce the blood flow in the circle of Willis. The next step included the occlusion of the left MCA. Operative access to the MCA was achieved via a trepanation hole in the left temporal bone. After trepanation, the dura mater was dissected, and the distal branches of the MCA were cauterised by electrocoagulation under an operating microscope, YZ-20T9 (Nanjing Redsun Optical Co., LTD, Nanjing, China). In the postoperative period, all animals received antibacterial (Ceftriaxone intramuscularly, 50 mg/kg once daily) and analgesic therapy (Ketorol intramuscularly, 2.5 mg/kg three times daily).

### 2.4. Experimental Groups

For the stroke gene therapy in the acute phase, GEL-VGN was prepared in the 14–16 h period before stroke modelling and was infused via the auricular vein 4 h after surgery. For the preventive gene therapy, GEL-VGN was prepared and infused 2 days before stroke modelling. In the study of the genetically modified WBCs expressing the reporter GFP homing in the brains of the experimental mini-pigs, two groups of animals were infused with GEL-GFP in the acute phase of stroke and before stroke modelling.

According to the design of the experiment, the animals were divided into 6 groups: (1) Con—the control group (*n* = 3, after stroke, animals were infused with saline), (2) TA—therapeutic acute group (*n* = 3, after stroke, animals were infused with GEL-VGN), (3) TP—therapeutic preventive group (*n* = 3, before stroke, animals were infused with GEL-VGN), (4) RA—reporter acute group (*n* = 2, after stroke, animals were infused with GEL-GFP), (5) RP—reporter preventive group (*n* = 2, before stroke, animals were infused with GEL-GFP), and (6) Int—intact group (*n* = 3, healthy animals were used to obtain basic molecular and immunofluorescence data for the comparative analysis with the results of experimental mini-pigs). In control and therapeutic groups, the animals were euthanised on day 21 after stroke modelling. The endpoint of the experiment was chosen based on studies of the recovery from arm disability within 3 weeks, observed in 80% of patients with stroke [17], and the 3-week period of adenoviral vector activity (effective expression of transgenes) [18].

### 2.5. Behavioural Tests

The “open field” test was used to evaluate the general locomotor activity levels of the experimental animals before and on days 3, 7, 14, and 21 after stroke modelling. The mini-pigs were placed in an arena (3 × 3 m) divided into 6 squares, and the horizontal activity was assessed by counting the number of lines crossed in 10 min, conducted by two supervisors in a blind manner in relation to the test animals.

### 2.6. Post-Ischaemic Changes in the Brain

#### 2.6.1. Sample Collection

At 21 days post-surgery, the control (Con) and treated animals (TA and TP) were anaesthetised, as described above, and were infused with potassium chloride (150 mg/kg) via the auricular vein. The brains were extracted from the cranial cavity and fixed in 4% paraformaldehyde solution. The brains of mini-pigs from the RA and RP groups were harvested 7 days after infusion with the GEL-GFP.

#### 2.6.2. Morphological Analysis of the Cerebral Infarction

To analyse the area of the infarction, digital images of the left hemispheres were captured. Afterwards, the whole brains were cut into 3 mm-thick frontal slices. The number of obtained slices varied from 4 to 7 and depended on the size of the ischemic injury area. For the morphometric analysis of the infarct volume, each 3 mm slice was photographed from both (frontal and caudal) sides. The absolute infarct volume (AIV) was calculated using digital images of each 3 mm-thick slice obtained from the infarct region in the left cerebral hemisphere. The relative infarct volume (RIV) was calculated as AIV/(AIV + V of brain). All morphometric analyses were performed using ImageJ software (NIH). Paraffin embedded brain tissue was cut with a rotary microtome HM 325 (Thermo Scientific, Waltham, WA, USA). The obtained frontal slices were stained with haematoxylin and eosin. For the histological analysis, digitised images of the brain cortex were taken with a microscope, Axioscope A1 (Carl Zeiss, Oberkochen, Germany).

#### 2.6.3. Homing of WBCs Expressing GFP in the Brain of Mini-Pigs

The migration of WBCs producing GFP in the brains was studied using fluorescent microscopy 7 days after intravenous the infusion of GML-GFP into the mini-pigs with stroke in the RA and RP groups. Samples from the left (peri-infarct region) hemisphere were fixed in 4% paraformaldehyde solution, cryoprotected in 30% sucrose, and embedded in tissue-freezing medium, and frozen 20 μm sections were prepared using a Microm HM 560 cryostat (Thermo Fisher Scientific, Waltham, WA, USA). Slide-mounted sections were incubated in DAPI solution (10 μg/mL in PBS; Sigma) and embedded in antifade mounting medium, and digital images were taken with a luminescence microscope (Axioscope A1, Carl Zeiss, Germany). GFP-positive WBCs were counted in 10 sections of the peri-infarct region of each mini-pig. The cell counting was performed based on digital images in an area of 600 × 500 μm. Average cell counts for each animal were standardised according to the area of 1 mm^2^.

#### 2.6.4. Immunofluorescence Staining

The immunofluorescence analysis of the molecular and cellular changes in the peri-infarct area was performed using the 20 μm slide-mounted sections, using primary antibodies (Ab) to specific cell markers (Table 1). The survivability of brain cells was evaluated using the Ab to pro-apoptotic protein (Caspase3) and heat shock protein 70 kDa (Hsp70). The expression of the synaptic proteins was assessed with the Abs against synaptic vesicle protein (Synaptophysin) and postsynaptic density protein 95 kDa (PSD95). The reaction of the neuroglial cells was evaluated using the Ab to glial fibrillary acidic protein (GFAP) for astrocytes, the Ab to oligodendrocyte transcription factor 2 (Olig2) for oligodendroglial cells, and the Ab to ionised calcium binding adaptor molecule 1 (Iba1) for microglial cells.

Appropriate secondary Abs were employed for an immune reaction with the primary Abs (Table 1). The cell nuclei were stained with DAPI (10 μg/mL in PBS, Sigma, St. Louis, MO, USA). Slices were embedded in glycerol (GalenoPharm, Saint Petersburg, Russia) and observed under a confocal LEICA TCS SP5 MP microscope (Leica Microsystems, Wetzlar, Germany). The images taken were used to analyse the counts of Caspase3 and Olig2 immunopositive nuclei (calculated taking into account the DAPI staining) and the immunopositive areas for Hsp70, Synaptophysin, PSD95, GFAP, and Iba1 (presented in percentages).

Regarding the quantitative evaluation, a total of 60 sections of the brain cortex were obtained by acquiring two adjacent 3 mm slices from the epicentre of the ischemic lesion from each animal. For the immunofluorescence staining, 6 sections were taken for each molecular marker. The immunoreactivity pattern of each target antigen in a single section was assessed over a total area of 0.5 mm^2^ using ImageJ software (NIH).

### 2.7. Analysis of Multiplex Cytokines, Chemokines, and Growth Factors 

The multiplex analysis was performed using the Porcine Cytokine/Chemokine MILLIPLEX^®^ magnetic-bead-based multi-analyte panel, containing the following target analytes: GM-CSF, IL-1ra, IL-2, IL-6, IL-10, IL-12, IL-18, TNFα, IFNγ, IL-1β, IL-1α, IL-4, and IL-8 (Millipore, Burlington, MA, USA). Blood and cerebrospinal fluid samples were obtained from the intact mini-pigs and each animal in the control and therapeutic TA and TP groups 3 weeks after stroke modelling and were appropriately processed, frozen at a temperature of −80 °C, and stored until the time of the study. The data were analysed using a MAGPIX^®^ system and the Analyst 5.1 software (Merck, Kenilworth, NJ, USA).

### 2.8. Statistical Analysis

The data processing, analysis, and visualisation were performed using R 4.1.0 (R Foundation for Statistical Computing, Vienna, Austria). Descriptive statistics are presented as the mean (standard deviation) for the behavioural tests and as the median (1st–3rd quartiles) for the morphometric analysis. The Kruskal–Wallis test, combined with Dunn’s test, as a post hoc method, was used for the group comparison. The results were visualized using box plots. Differences were considered statistically significant when *p* < 0.05.

## 3. Results

### 3.1. Expression of the Reporter and Therapeutic Genes in WBCs In Vitro

The efficacy of the leucoconcentrate transduction with the chimeric Ad5/35 vector was confirmed for the samples of GEL-GFP and GEL-VGN 72 h after the cultivation of WBCs in vitro by flow cytometry and the RT-PCR methods, as we described recently [13]. The obtained results are consistent with the previously obtained data. In the GEL-GFP samples, the flow cytometry revealed 1.5 ± 2.7% of the GFP-positive WBC. In the GEL-VGN samples, the RT-PCR analysis demonstrated the increase in the mRNA levels for vegf165 by 80.6 ± 18.7 times, for gdnf by 152.6 ± 50.3 times, and for ncam1 by 43.5 ± 5.6 times in comparison with the non-transduced WBCs. The data are presented as mean ± SD.

### 3.2. Locomotor Activity

**Figure 2:**
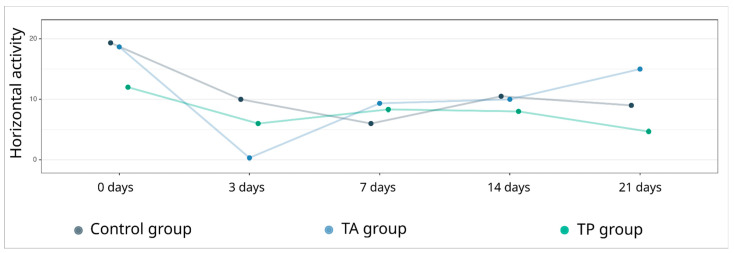
“Open field” test. Analysis of the horizontal activity of the experimental animals before stroke modelling on day 0 and 3, 7, 14, and 21 days after surgery.

The base results, presented as the mean (standard deviation) in the control (19.3 (16.6)) and therapeutic TA (18.7 (16.3)) and TP (12.0 (5.6)) groups, were obtained during the training of the animals before surgery (Figure 2). On day 3, after the stroke modelling, there was a decrease in the motor activity in all groups, with subsequent recovery by days 7 and 14. On the 21st day, the most improved performance was revealed in the TA (15.0 (8.7)) and TP (4.7 (1.5)) therapeutic groups, compared with the control group (9.0 (5.5)), corresponding to the base data. It is important to note that because very few animals were used in the behavioural test, the data are not sufficient enough to provide an acceptable level of power; therefore, the results regarding the locomotor activity should be considered exploratory due to the insufficient strength of the evidence.

### 3.3. Morphological Analysis of the Infarction Region

#### 3.3.1. Infarction Volume

**Figure 3:**
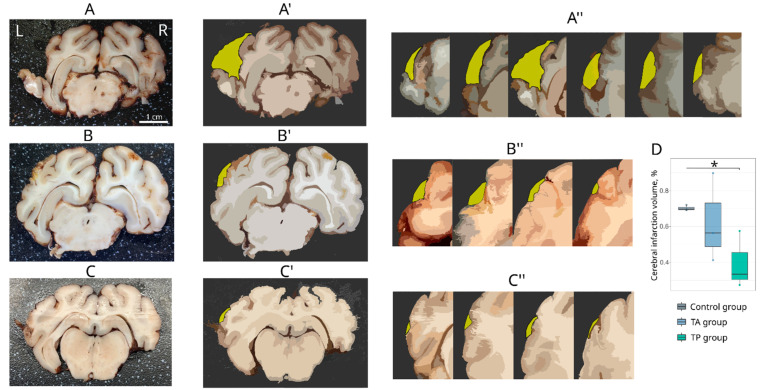
Infarction volume analysis. (**A**–**C**) Images of 3 mm thick frontal slices of the brain from the area of ischaemic damage: (**A**) control mini-pigs 21 days after stroke modelling; (**B**) mini-pigs treated with the autologous genetically enriched leucoconcentrate 4 h after stroke modelling (TA group); (**C**) mini-pigs treated with the autologous genetically enriched leucoconcentrate 2 days before stroke modelling (TP group). (**A**′,**B**′,**C**′) Reconstruction of the brain infarction area (minus tissue) in the left hemisphere (coloured yellow). (**A**″,**B**″,**C**″) Reconstructed infarction area based on complete sets of 3 mm slices from frontal to caudal direction for each experimental group. (**D**) Morphometric analysis of cerebral infarction volume, * *p* < 0.05. L—left hemisphere, R—right hemisphere. The scale in (**A**) corresponds to that in (**B**,**C**).

In all the experimental mini-pigs, the macroscopic examination of the left hemisphere 21 days after the MCA occlusion showed a focal ischemic infarction localized in the temporal lobe (Figure 3A–C). Vasodilation was observed in the right hemisphere of these animals. In the control animals, the infarct volume, presented as the median (1st–3rd quartiles), was 0.7 (0.7–0.7). In the treated mini-pigs, the infarct volume in the TA group was 0.6 (0.5–0.7) and two times lower in the TP group (0.3 (0.3–0.5), *p* = 0.0526) in comparison with the control animals (Figure 3D).

#### 3.3.2. Histological Study

**Figure 4:**
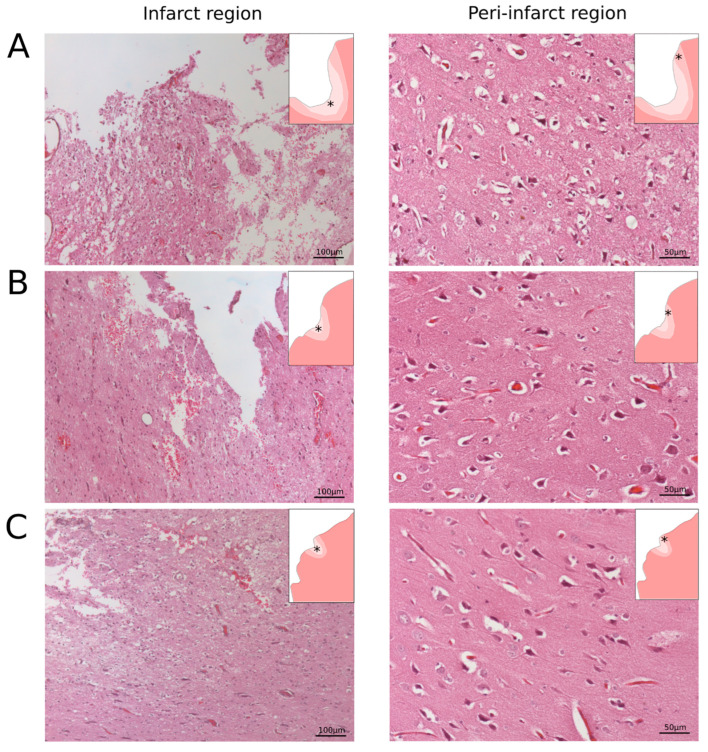
Frontal slices of the cerebral cortex from the peri-infarction area stained with haematoxylin and eosin. (**A**) Control mini-pigs 21 days after stroke modelling; (**B**) mini-pigs treated with autologous genetically enriched leucoconcentrate 4 h after stroke modelling (TA group); (**C**) mini-pigs treated with autologous genetically enriched leucoconcentrate 2 days before stroke modelling (TP group). The asterisks in the schematic fragment of the cerebral cortex with the ischemic lesion correspond to the areas of the histological sections.

The microscopic study of the haematoxylin- and eosin-stained frontal sections of the cerebral cortex in the control group showed crater-shaped lesions. Reactive changes in the adjacent pia mater were noted in the form of infiltration (predominantly mononuclear), fibrosis, and haemosiderosis. In the crater area, the cortex structure was disturbed, and there were no neurons, while the brain tissue showed areas of tissue looseness, with microcystic changes, “foamy” cells, microcirculatory disorders in the form of clots in the capillaries, and extravasations. There were also post-necrotic changes related to the denudation of the blood vessels. In the peri-infarct region, pronounced changes in the neurons in the form of pyknosis, hyperchromia, and basophilia with barely noticeable cell structures, as well as a lack of neurites, were detected (Figure 4A). In contrast to the control group, in the therapeutic TA group treated in the acute phase, less pronounced foci of destruction, microcirculatory changes, and the absence of infiltration in the crater area were identified. In the peri-infarct region, pyknotic neurons were detected, but many of them had visible nuclei with nucleoli inside and the presence of neurites (Figure 4B). In the TP group treated with preventive therapy, the foci of damage were significantly smaller in size, and there were almost no foci of destruction in the crater area. In contrast to the control and TA groups described above, single neurons with pyknotic changes but clearly defined processes and non-prominent tissue looseness with minimal microcystic changes were demonstrated. Reactive infiltrative changes were observed in the adjacent pia mater. A few pyknotic neurons with clear, visible neuritis were found in the peri-infarct region (Figure 4C).

### 3.4. Immunofluorescence Analysis of the Peri-Infarct Region

#### 3.4.1. Cellular Stress Response and Apoptosis

**Figure 5:**
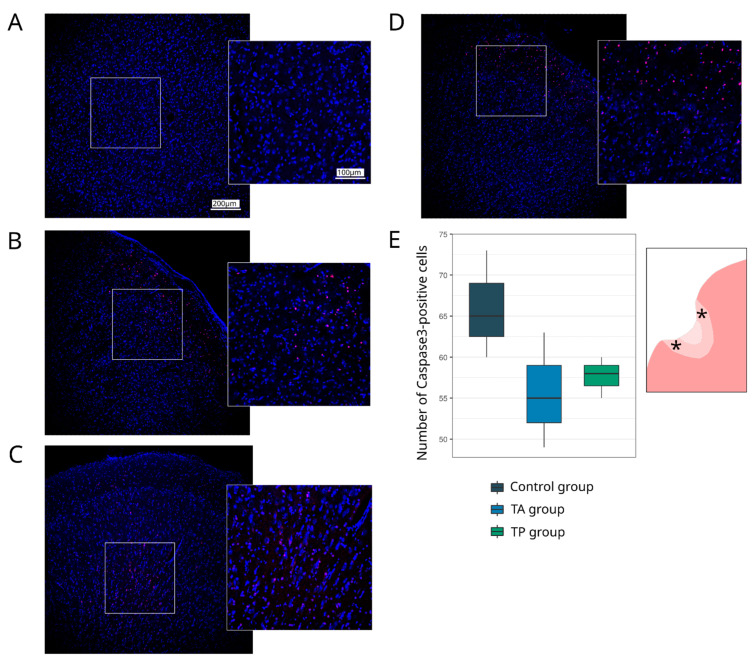
Expression of a pro-apoptotic protein. Immunofluorescence staining of the brain cortex in the peri-infarct region with antibodies against Caspase3 (red). Nuclei were counterstained with DAPI (blue). (**A**) Intact mini-pigs; (**B**) control mini-pigs 21 days after stroke modelling; (**C**) mini-pigs treated with the autologous genetically enriched leucoconcentrate 4 h after stroke modelling (TA group); (**D**) mini-pigs treated with the autologous genetically enriched leucoconcentrate 2 days before stroke modelling (TP group); (**E**) box plots demonstrate the number of Caspase3-positive cells in the experimental groups, The scale of the images in (**A**) corresponds to that in (**B**–**D**). The asterisks in the schematic fragment of the cerebral cortex with the ischemic lesion indicate the areas used for the immunofluorescence analysis.

**Figure 6:**
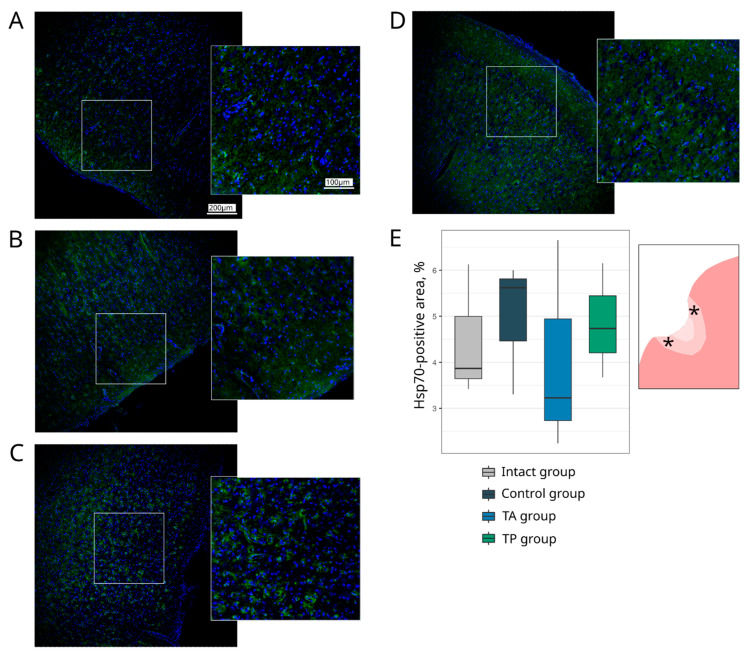
Expression of a cellular stress response protein. Immunofluorescence staining of the brain cortex in the peri-infarct region with the antibodies against heat shock protein 70 kDa (Hsp70) (green). Nuclei were counterstained with DAPI (blue). (**A**) Intact-mini pigs; (**B**) control mini-pigs 21 days after stroke modelling; (**C**) mini-pigs treated with the autologous genetically enriched leucoconcentrate 4 h after stroke modelling (TA group); (**D**) mini-pigs treated with the autologous genetically enriched leucoconcentrate 2 days before stroke modelling (TP group); (**E**) box plots demonstrate the mean (%) of the Hsp70-positive area in the intact and experimental groups. The scale of the images in (**A**) corresponds to that in (**B**–**D**). The asterisks in the schematic fragment of the cerebral cortex with the ischemic lesion indicate the areas used for the immunofluorescence analysis.

Cell markers for apoptosis (Caspase3) and for heat shock protein 70 kDa (Hsp70) were used to analyse the brain cell survivability (Figure 5). The count of Caspase3-positive cells in the control group revealed 65.00 (62.50–69.00) apoptotic cells, which was more than the number observed in the TA (55.00 (52.00–59.00), *p* = 0.0712) and in the TP (58.00 (56.50–59.00), *p* = 0.1145) groups. The Hsp70-positive relative area did not differ between the intact and experimental groups (Figure 6).

#### 3.4.2. Synaptic Proteins Expression

**Figure 7:**
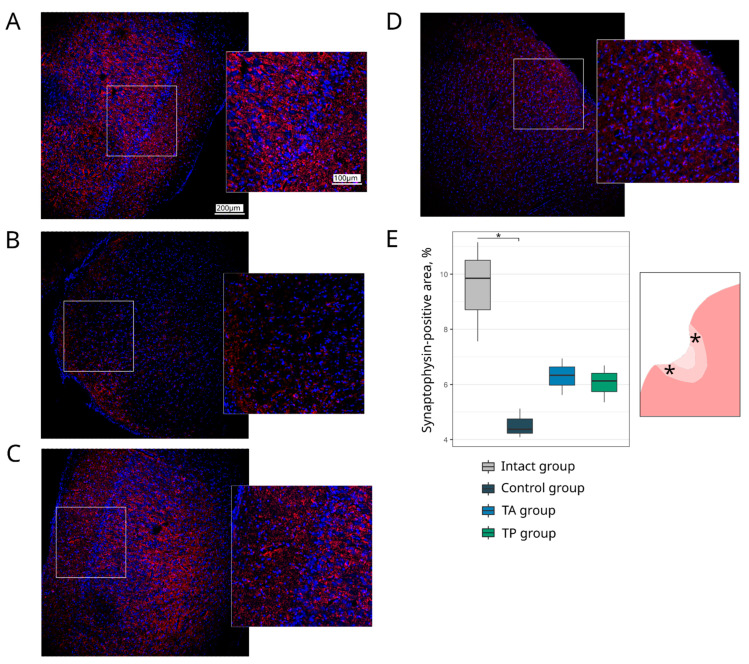
Expression of a synaptic vesicle protein. Immunofluorescence staining of the brain cortex in the peri-infarct region with antibodies against Synaptophysin (red). Nuclei were counterstained with DAPI (blue). (**A**) Intact-mini pigs; (**B**) control mini-pigs 21 days after stroke modelling; (**C**) mini-pigs treated with the autologous genetically enriched leucoconcentrate 4 h after stroke modelling (TA group); (**D**) mini-pigs treated with the autologous genetically enriched leucoconcentrate 2 days before stroke modelling (TP group); (**E**) box plots demonstrate the mean (%) of the Synaptophysin-positive area in the intact and experimental groups, * *p* < 0.05. The scale of the images in (**A**) corresponds to that in (**B**–**D**). The asterisks inserted in the schematic fragment of the cerebral cortex with the ischemic lesion indicate the areas used for the immunofluorescence analysis.

**Figure 8:**
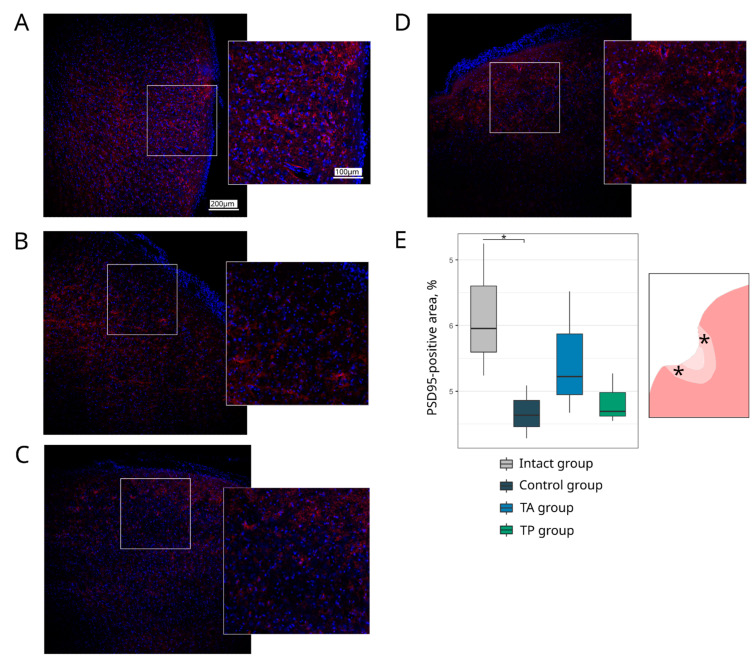
Expression of a post-synaptic density protein. Immunofluorescence staining of the brain cortex in the peri-infarct region with antibodies against post-synaptic density protein 95 kDa (PSD95) (red). Nuclei were counterstained with DAPI (blue). (**A**) Intact-mini pigs; (**B**) control mini-pigs 21 days after stroke modelling; (**C**) mini-pigs treated with autologous genetically enriched leucoconcentrate 4 h after stroke modelling (TA group); (**D**) mini-pigs treated with autologous genetically enriched leucoconcentrate 2 days before stroke modelling (TP group); (**E**) box plots demonstrate the mean (%) of the PSD95-positive area in the intact and experimental groups, * *p* < 0.05. The scale of the images in (**A**) corresponds to that in (**B**–**D**). The asterisks in the schematic fragment of the cerebral cortex with the ischemic lesion indicate the areas used for the immunofluorescence analysis.

To evaluate the functional recovery of the neurons in the post-ischaemic brains, we investigated the expression of the synaptic vesicle protein Synaptophysin and post-synaptic density protein 95 kDa (PSD95). In the peri-infarct region, in the control group, the Synaptophysin-immunopositive area was decreased (4.37 (4.23–4.75), *p* = 0.0022) compared with the intact group (9.85 (8.71–10.50)). In the therapeutic groups, in both TA (6.33 (5.97–6.64)) and TP (6.12 (5.74–6.41)), the expression of the Synaptophysin did not differ from the intact group (Figure 7). The PSD95-immunipositive area was decreased in the control group (4.63 (4.46–4.86), *p* = 0.0235) and did not differ in the TA (5.22 (4.95–5.87)) and TP (4.69 (4.62–4.98)) groups in comparison with the intact group (5.95 (5.60–6.60)) (Figure 8).

#### 3.4.3. Neuroglia Remodelling

**Figure 9:**
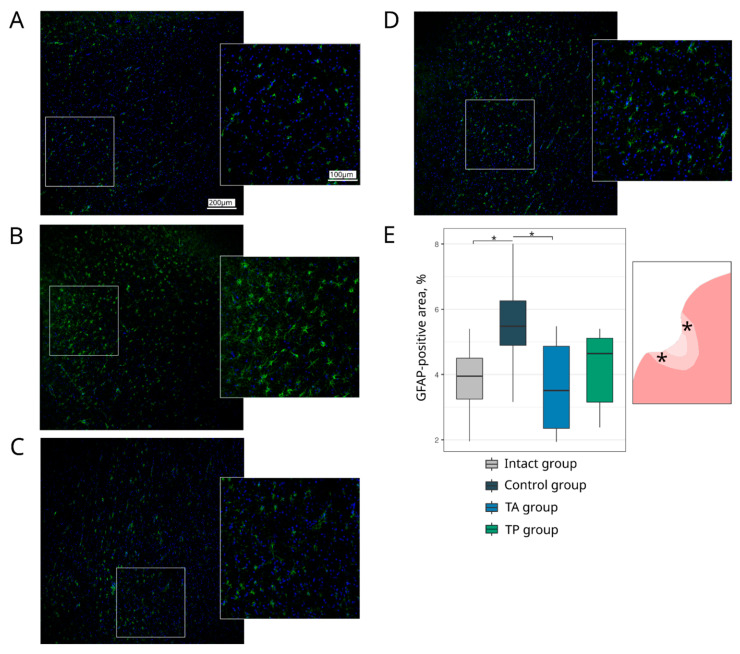
Expression of a glial fibrillary acidic protein. Immunofluorescence staining of the brain cortex in the peri-infarct region with antibodies against glial fibrillary acidic protein (GFAP), used to identify astrocytes (green). Nuclei were counterstained with DAPI (blue). (**A**) Intact-mini pigs; (**B**) control mini-pigs 21 days after stroke modelling; (**C**) mini-pigs treated with the autologous genetically enriched leucoconcentrate 4 h after stroke modelling (TA group); (**D**) mini-pigs treated with the autologous genetically enriched leucoconcentrate 2 days before stroke modelling (TP group); (**E**) box plots demonstrate the mean (%) of the GFAP-positive area in the intact and experimental groups, * *p* < 0.05. The scale of the images in (**A**) corresponds to that in (**B**–**D**). The asterisks in the schematic fragment of the cerebral cortex with the ischemic lesion indicate the areas used for the immunofluorescence analysis.

**Figure 10:**
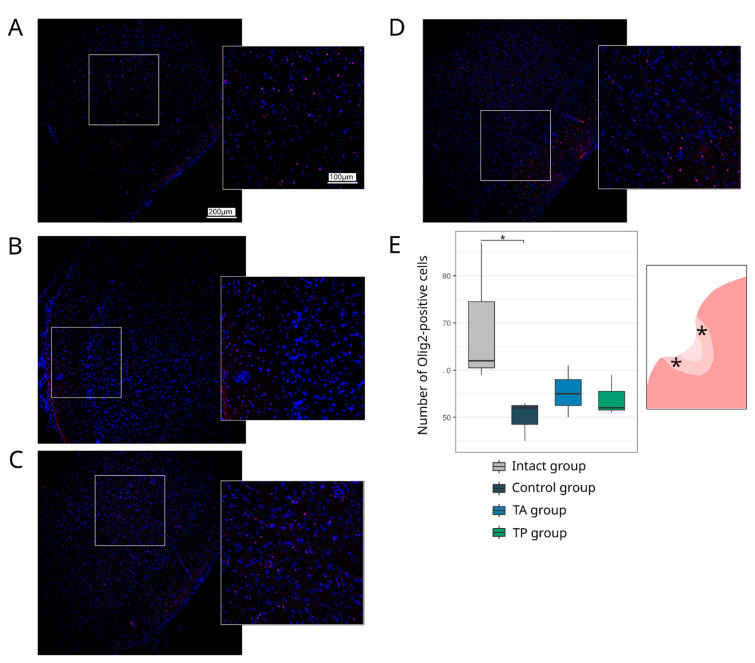
Expression of an oligodendrocyte transcription factor. Immunofluorescence staining of the brain cortex in the peri-infarct region with antibodies against oligodendrocyte. Transcription factor 2 (Olig2) was used to identify oligodendroglial cells (red). Nuclei were counterstained with DAPI (blue). (**A**) Intact-mini pigs; (**B**) control mini-pigs 21 days after stroke modelling; (**C**) mini-pigs treated with the autologous genetically enriched leucoconcentrate 4 h after stroke modelling (TA group); (**D**) mini-pigs treated with the autologous genetically enriched leucoconcentrate 2 days before stroke modelling (TP group); (**E**) box plots demonstrate the number of Olig2-positive nuclei in the intact and experimental groups, * *p* < 0.05. The scale of the images in (**A**) corresponds to that in (**B**–**D**). The asterisks in the schematic fragment of the cerebral cortex with the ischemic lesion indicate the areas used for the immunofluorescence analysis.

**Figure 11:**
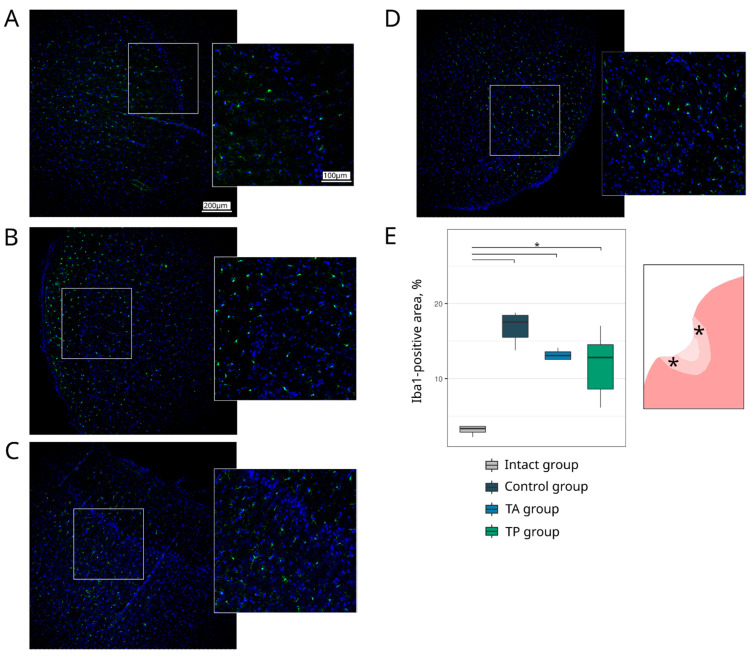
Expression of an ionized calcium binding adaptor molecule 1. Immunofluorescence staining of the brain cortex in the peri-infarct region with antibodies against ionized calcium binding adaptor molecule 1 (Iba1), used to identify microglial cells (green). Nuclei were counterstained with DAPI (blue). (**A**) Intact mini-pigs; (**B**) control mini-pigs 21 days after stroke modelling; (**C**) mini-pigs treated with the autologous genetically enriched leucoconcentrate 4 h after stroke modelling (TA group); (**D**) mini-pigs treated with the autologous genetically enriched leucoconcentrate 2 days before stroke modelling (TP group); (**E**) box plots demonstrate the number of Iba1-positive nuclei in the intact and experimental groups, * *p* < 0.05. The scale of the images in (**A**) corresponds to that in (**B**–**D**). The asterisks inserted into the schematic fragment of the cerebral cortex with the ischemic lesion indicate the areas used for the immunofluorescence analysis.

Inflammation, astrogliosis, and demyelination are the main pathological events that take place in the peri-infarct region, where the progression of each correlates with the volume of the ischaemic infarct. In this study, the remodelling of astrocytes, oligodendroglial cells, and microglial cells in the peri-infarct region was investigated using the specific Abs to GFAP, Olig2, and Iba1, respectively. The analysis of the GFAP-positive astrocytes revealed the increase in the GFAP-positive area in the control group (5.48 (4.89–6.26)) in comparison to the intact group (3.95 (3.25–4.50), *p* = 0.0453) and TA (3.51 (2.35–4.86), *p* = 0.0284) groups. In the therapeutic TA and TP (4.64 (3.16–5.11)) groups, the GFAP-positive area did not differ from the intact group (Figure 9). The number of myelin-forming Olig2-positive cells was reduced in the control group (52.00 (48.50–52.50), *p* = 0.0231) relative to the intact group (62.00 (60.50–74.50)). The numbers of Olig2-positive cells in the therapeutic TA (55.00 (52.50–58.00)) and TP (52.00 (51.50–55.50)) groups were comparable with the intact group (Figure 10). The analysis of the Iba1-positive microglial cells demonstrated the increase in the Iba1-positive areas, corresponding to the activated microglial cells in the control (17.53 (15.50–18.44), *p* < 0.0001), TA (13.09 (12.55–13.57), *p* = 0.0014), and TP (12.84 (8.61–14.51), *p* = 0.0033) groups relative to the intact group (3.35 (2.85–3.64)). However, the area occupied by the activated microglia was less prominent in the therapeutic groups than in the control group (Figure 11).

### 3.5. Homing of GFP-Positive WBCs in the Brain

**Figure 12:**
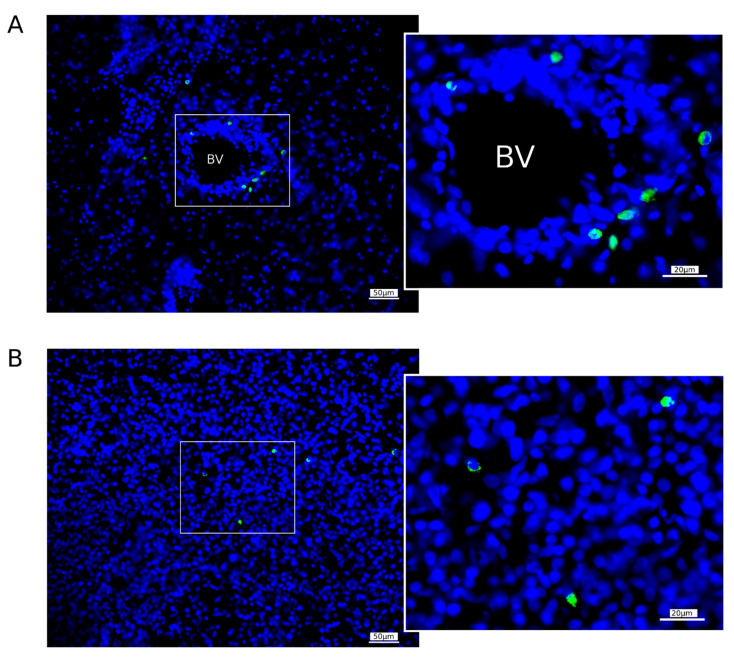
Expression of a green fluorescence protein in the brain one week after the intravenous infusion of the autologous leucoconcentrate enriched with the GFP gene. (**A**) Mini-pigs infused with autologous genetically enriched leucoconcentrate 4 h after stroke modelling (RA group); (**B**) mini-pigs infused with autologous genetically enriched leucoconcentrate 2 days before stroke modelling (RP group). A specific green glow of GFP is observed in the WBCs transduced with GFP gene. Nuclei were counterstained with DAPI (blue). BV—blood vessel.

A week after the intravenous infusion of the autologous GEL-GFP into the mini-pigs 4 h after stroke modelling (RA group) and 2 days before surgery (RP group), the 20 μm-thick sections from the peri-infarct region in the left hemisphere were studied using fluorescent microscopy. Using the green channel GFP-positive cells, an intense green fluorescence was observed in all the studied sections (Figure 12). Counterstaining with DAPI revealed the co-localisation of the nuclei with GFP fluorescence. The size of the cells and the round-shaped nuclei correspond to lymphocytes and monocytes. A few GFP-positive WBCs were found in both the RA (26.1 (15) cells/mm^2^) and RP ((7.4 (8.5) cells/mm^2^) groups. These data support our hypothesis about the ability of genetically modified WBCs, after intravenous infusion, to migrate to the area of ischaemic injury and produce recombinant molecules, which, by a paracrine mechanism, enhances the tissue neuroplasticity in the peri-infarct region and has a positive effect on the affected nerve tissue remodelling.

### 3.6. Cytokine, Chemokine, and Growth Factor Profiling in the Blood and Cerebrospinal Fluid

**Figure 13:**
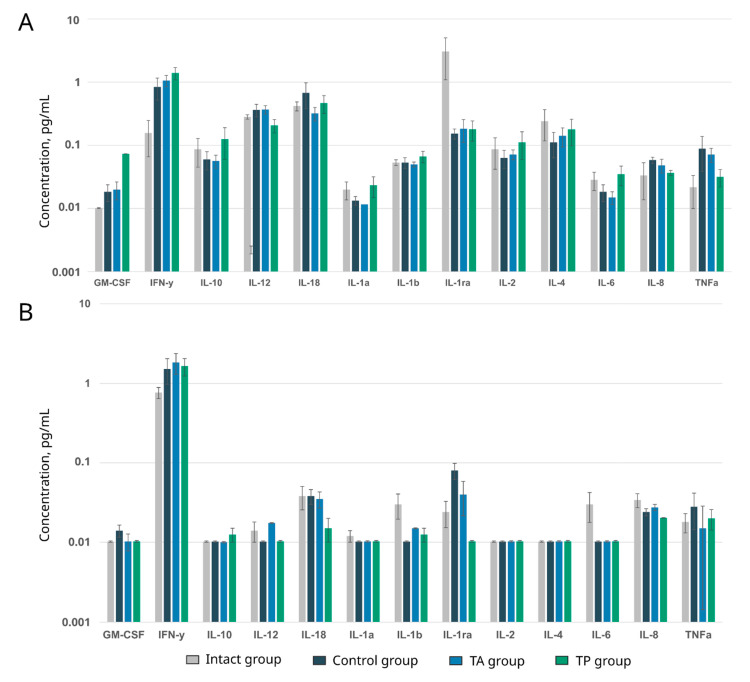
Multiplex profiling of cytokines, chemokines, and growth factors in the blood (**A**) and cerebrospinal fluid (**B**) of intact mini-pigs, control mini-pigs 21 days after stroke modelling, mini-pigs treated with the autologous genetically enriched leucoconcentrate 4 h after stroke modelling (TA group), and mini-pigs treated with the autologous genetically enriched leucoconcentrate 2 days before stroke modelling (TP group). Differences between the analysed groups were assessed by one-way ANOVA, with a *p* value of <0.05 being considered as statistically significant.

Inflammatory mediators produced by activated microglia, astrocytes, and endothelial cells are important contributors to the progression of ischemic brain injuries [19]. Local and systemic immune responses play a significant role not only in the primary ischemic damage but also in the weeks following post-ischemic brain remodelling [20]. A simultaneous analysis of GM-CSF, IL-1ra, IL-2, IL-6, IL-10, IL-12, IL-18, TNFα, IFNγ, IL-1β, IL-1α, IL-4, and IL-8 in the blood and cerebrospinal fluid did not identify prominent differences in the rates of the studied inflammatory mediators in all the samples (Figure 13). Thus, the contents of key pro-inflammatory (IL-1, IL-6, and TNF-α) and anti-inflammatory (IL-10, IL-12) cytokines in the blood and cerebrospinal fluid in the control and treated mini-pigs were equal when compared to the intact animals. These data are in line with our previous results obtained using rats [11] and demonstrate the termination of the inflammatory response 3 weeks after stroke modelling. Additionally, a comparative analysis showed that GEL-VGN had no effect on the levels of inflammatory mediators in the blood and cerebrospinal fluid of the treated animals. However, the higher levels of IL-1ra (an inhibitor of the pro-inflammatory effect of IL1β) in the blood and IL-6 (a neuropoietic cytokine) in the cerebrospinal fluid in intact animals, compared to the experimental mini-pigs, may indicate the incomplete recovery of the animals 3 weeks after stroke modelling.

## 4. Discussion

Various viral vectors are used to deliver therapeutic genes to the CNS [21]. However, the problems of mutagenesis, immunogenicity, and the duration of gene expression in direct (in vivo) gene therapy remain relevant to this day. For the temporary expression of transgenes, the cell-mediated approach to gene delivery is considered an effective and safe method of gene therapy. In stroke gene therapy, for the delivery of the recombinant neurotrophic factors to the ischaemic brain, mesenchymal stem cells [22,23,24] and neural stem cells [25] have been used. In our previous studies on the rat MCA occlusion model, we employed ex vivo gene-engineered umbilical cord blood mononuclear cells (UCB-MC), simultaneously transduced with three adenoviral vectors (Ad5) carrying *vegf165, gdnf,* and *ncam1*, for cell-mediated gene therapy in the acute phase [10] and for the preventive treatment [11] of stroke. Our results demonstrated the positive effect of the intrathecal injection of UCB-MC, expressing recombinant VEGF, GDNF, and NCAM, on the reduction in the negative consequences of ischaemia-induced brain injury. Recently, for the delivery of the same therapeutic gene combination to the mini-pig post-traumatic spinal cord, for the first time, we used an intravenous administration of the autologous GEL as a complementary therapy to the supra- and sub-lesional epidural electrical stimulation [13]. The study demonstrated the prospects of using genetically enriched leucoconcentrate for the stimulation of neuro-regeneration in the CNS. The rationale for the preparation of the autologous, genetically enriched leucoconcentrate for cell-mediated gene therapy is based on its simplicity and biosafety. In the current study, we employed this strategy for stroke treatment in the acute phase and for preventive gene therapy in a mini-pig stroke model.

To test the utility of the autologous, genetically enriched leucoconcentrate for stroke gene therapy, we developed an original, reliable, and reproducible model of ischaemic stroke in mini-pigs based on previously reported methods. In the study, we hypothesized two possible mechanisms for the positive effect of GEL-VGN on the post-ischemic brain recovery: (1) genetically modified WBCs can pass through the disrupted blood–brain barrier [26] and occupy the ischemic region, so that secreted recombinant VEGF, GDNF, and NCAM can act on the target cells locally through a paracrine mechanism; and (2) recombinant VEGF, GDNF, and NCAM secreted by the genetically modified WBCs into the bloodstream can diffuse through the leaky blood–brain barrier into the brain tissue and modulate its neuroplasticity through an endocrine mechanism.

The model of focal cerebral infarction in mini-pigs, using the method of the occlusion of the MCA through the transorbital approach, was examined for the first time by Sakoh et al. [27]. Imai et al. were the first to use a craniotomy to access the MCA instead of a transorbital approach [28]. In this study, we combined the occlusion of the distal branches of the MCA via a trepanation hole with the occlusion of the right common carotid artery to reduce the blood flow in the circle of Willis and observed a reproducible focus of ischemic cerebral cortex infarction in the temporal lobe of the left hemisphere, accompanied by moderate locomotor dysfunction. At day 21 after surgery, an immunofluorescence study of the peri-infarct region revealed apoptotic cells; an increase in the area occupied by microglia and astrocytes, which eventually leads to the formation of a glial scar; a decrease in the number of oligodendroglial cells responsible for myelination; and the decrease in the synaptic protein expression by neural cells. The intravenous infusion of the autologous leucoconcentrate enriched with *vegf165*, *gdnf*, and *ncam1* genes into the mini-pigs in the acute phase of the stroke or 2 days before the modelling of the stroke (preventive gene therapy) resulted in the positive remodelling of the peri-infarct region, including: (1) a smaller infarct volume and number of apoptotic cells; (2) restraining of the organisation of the glial scar; (3) a higher number of myelin-forming oligodendroglial cells; and (4) the restoration of the functional activity of neurons, according to the levels of the synaptic vesicle protein Synaptophysin and postsynaptic density protein (PSD95) expression. The results of the cytokine, chemokine, and growth factor profiling of the blood and cerebrospinal fluid in the experimental mini-pigs should also be considered positive. We found no difference in the levels of main pro-inflammatory (IL-1, IL-6, and TNF-α) and anti-inflammatory (IL-10, IL-12) cytokines in the blood and cerebrospinal fluid of the control and treated mini-pigs when compared with the intact animals. These data demonstrate the termination of the inflammatory response 3 weeks after stroke modelling, although the higher contents of IL-1ra (proinflammatory inhibitor IL1β) in the blood and IL-6 (neuropoietic cytokine) in the cerebrospinal fluid in the intact animals, compared to the experimental mini-pigs, show the incomplete recovery of the animals with stroke. In addition, the similar levels of investigated cytokines, chemokines, and growth factors in the control and treated animals may indicate the absence of immunogenic effects of the genetically modified WBCs at the local and systemic levels.

One of the important issues regarding the strategy of using gene therapy for stroke is the targeted delivery of recombinant genes to the site of ischemic damage. WBCs are a cell type with a high synthetic and secretory activity and the ability to migrate successfully from the bloodstream to the affected tissues. In the study, we demonstrated the potential of genetically modified WBCs to pass through the disrupted blood–brain barrier and occupy the ischemic area. The presence of the gene-modified WBCs expressing GFP in the peri-infarct region of the experimental mini-pigs one week after the administration of the GEL-GFP in the acute phase of stroke or 2 days before the stroke modelling supports our hypothesis, stating that after intravenous infusion of the autologous GEL-VGN, gene-modified WBCs migrate to the brain ischaemic injury region and locally produce the recombinant therapeutic molecules. In the acute phase of stroke, the compromised blood–brain barrier leads not only to the migration of gene-modified WBCs but is also permeable and subject to the infusion of recombinant VEGF, GDNF, and NCAM secreted by gene-modified WBCs into the bloodstream. Thus, the positive effect of GEL-VGN on post-ischemic brain recovery may be due to the action of the therapeutic molecules through paracrine and endocrine mechanisms. VEGF and GDNF have known neuroprotective properties and are widely used in gene therapy studies of neurodegenerative diseases, neurotrauma, and stroke. It is assumed that NCAM plays a role not only as a stimulator of regeneration [29] but also in ensuring the homing and survival of genetically modified leucocytes in the brain tissue. Due to the lack of ability of the adenoviral vector to integrate into the recipient’s genome, the temporary secretion of the recombinant VEGF, GDNF, and NCAM continues for approximately 3 weeks [18].

The issue of stroke prevention in patients at high risk of ischemic stroke, combined with transient ischemic attacks of different origins, requires constant clinical examination and treatment with anticoagulants. Our concept of preventive personalized gene therapy is based on the intravenous infusion of GEL-VGN in patients at high risk of stroke. An autologous leucoconcentrate, enriched with genetic material, may be prepared in hospital blood centres using a routine unit of a patient’s peripheral blood and chimeric adenoviral vectors (Ad5/35) carrying recombinant therapeutic genes. Importantly, the procedure for preparing the genetically enriched leucoconcentrate is performed in a standard blood bag, without the use of any animal products, antibiotics, or other chemicals for culturing cells in vitro. This approach can reduce the mass death of neurons in the ischemic penumbra area during the 3–6 h period of the therapeutic window at the onset of stroke, and it could become an important component of emergency therapy [30].

For the first time, in this study, we demonstrated a new approach to the use of personalized ex vivo gene therapy for stroke based on a simple, economical, and safe platform for the delivery of the recombinant therapeutic gene/genes using an autologous leucoconcentrate, prepared from the patient’s peripheral blood. The transduction of WBCs to the leucoconcentrate was performed in a blood container using a chimeric adenoviral vector (Ad5/35), which has a modified fibre with a high affinity for the CD46 membrane molecule, expressed in all WBCs [31], without culturing in vitro and without any risks stemming from the use of animal-origin bioproducts and antibiotics.

Other important issues regarding stroke therapy include the development of treatments under the threat of stroke. Preventive gene therapy can restrain ischemic brain damage after the onset of a stroke. The presence of the recombinant neuroprotective molecules can increase the survivability of neurons in the ischemic area immediately after the onset of a stroke. The results obtained in the study confirmed our hypothesis of the rationality of preventive gene therapy based on the intravenous infusion of autologous GEL-VGN under the threat of stroke.

A comparative analysis of the present results and data from our previous studies on triple-gene therapy for stroke in rats with MCO, using the intrathecal injection of human genetically engineered UCB-MC expressing recombinant molecules VEGF, GDNF, and NCAM in the acute phase [10] and for preventive treatment [11], revealed similar effects of gene therapy on the cortical remodelling in rats and mini-pigs. These data demonstrate, on the one hand, the consistency of the findings on the efficacy of triple-gene therapy for stroke treatment in small and large animals and, on the other hand, the efficacy of the intravenous infusion of autologous genetically modified WBCs. Thus, based on our previous investigations and the present study, we propose a new strategy for personalized cell-mediated gene therapy for stroke treatment based on the intravenous infusion of the autologous GEL temporally secreting neuroprotective factors (VEGF, GDNF, and NCAM).

## 5. Conclusions

In the current study, we demonstrated that the occlusion of the distal branches of the MCA, in combination with the occlusion of the right common carotid artery, in mini-pigs results in a reproducible focal ischaemic cerebral infarction in the temporal lobe of the left hemisphere. Triple-gene therapy in the acute phase and for the preventive treatment of stroke in a mini-pig model, using the approach of the intravenous infusion of the autologous genetically enriched leucoconcentrate producing recombinant neuroprotective molecules (VEGF, GDNF and CAN), demonstrated beneficial effects on the preservation and recovery of the brain after ischemic stroke. Thus, the employment of the autologous WBCs enabling the temporary production of recombinant therapeutic molecules to correct the pathological process in the CNS may be one of the breakthrough approaches in gene therapy.

## Figures and Tables

**Table 1:** Antibodies used for immunofluorescence study. Reprinted from Ref. [13].

Antibody Against	Host	Dilution	Source
Caspase3	Rabbit	1:200	Cell Signalling Technology (Danvers, MA, USA) (Cat 9661)
Glial fibrillary acidic protein (GFAP)	Mouse	1:200	Santa Cruz (Santa Cruz, CA, USA) (Cat sc-33673)
Ionized calcium binding adaptor molecule 1 (Iba1)	Rabbit	1:150	Abcam (Cambridge, UK) (Cat ab178847)
Heat shock protein 70 kDa (Hsp70)	Rabbit	1:200	Abcam (Cat ab239732)
Oligodendrocyte transcription factor 2 (Olig2)	Rabbit	1:100	Abcam (Cat ab220796)
Postsynaptic density protein 95 kDa (PSD95)	Rabbit	1:200	Abcam (Cat ab18258)
Synaptophysin	Rabbit	1:100	Abcam (Cat ab32127)
Mouse IgG conjugated with Alexa 488 *	Donkey	1:200	Invitrogen (Carlsbad, CA, USA) (Cat A-21202)
Rabbit IgG conjugated with Alexa 488 *	Donkey	1:200	Invitrogen (Cat A-21206)
Rabbit IgG conjugated with Alexa 647 *	Donkey	1:200	Invitrogen (Cat A-31573)

*—secondary antibodies.
